# Market of tissue engineering in Canada from 2011 to 2020

**DOI:** 10.3389/fbioe.2023.1170423

**Published:** 2023-05-31

**Authors:** Ning Tate Cao, Subhiksha Muthukumaran, Xiongbiao Chen

**Affiliations:** ^1^ Ron and Jane Graham School of Professional Development, University of Saskatchewan, Saskatoon, SK, Canada; ^2^ Department of Mechanical Engineering, University of Saskatchewan, Saskatoon, SK, Canada; ^3^ Division of Biomedical Engineering, University of Saskatchewan, Saskatoon, SK, Canada

**Keywords:** tissue engineering, bioprinting, regenerative medecine, canadian market, stem cells

## Abstract

Tissue engineering aims to produce tissue/organ substitutes to improve upon current treatment approaches, thus providing a permanent solution to damaged tissues/organs. This project aimed to perform a market analysis for understanding and promoting the development and commercialization of tissue engineering in Canada. We searched companies that were established between October 2011 and July 2020 via publicly available information and for these companies, we collected and analyzed the corporate level information, including revenues, and number of employees and founder information. The companies assessed were mainly searched from four different industry segments, i.e., bioprinting, biomaterials, cells and biomaterials, and stem-cells related industry. Our results have demonstrated that there are twenty-five tissue-engineering companies registered in Canada. These companies generated an estimated revenue of USD $67 million in the year 2020, most generated by the tissue engineering and stem-cells related industries. Our results also show that Ontario has the largest number of headquarters of tissue engineering companies among the provinces or territories of Canada. It is expected that the number of new products undergoing clinical trials is increased, based on our results of current clinical trials. Altogether, tissue engineering in Canada has shown a huge growth in the past decade and is forecasted to be an emerging industry in Canada for the years to come.

## 1 Introduction

Millions of people suffer from tissue/organ injuries or damage, such as peripheral nerve injuries and heart attacks, and need treatments. In Canada, 130,000 surgeries/year are performed in Canada to replace hips/knees, and at the end of 2018 some 4,351 people were on waiting lists for organ transplantation, 223 of whom died (Cihi, 2023). Tissue engineering (TE) aims to produce tissue/organ substitutes or scaffolds to improve upon current treatment approaches, thus providing a permanent solution to tissues/organs injuries (Griffith and Naughton, 2002), (Griffith and Naughton, 2002). An analogy would be buying new parts at the mechanic to replace car parts that are broken or no longer functioning. Success in TE means someone who suffers a tissue/organ injury could go to a hospital, have the engineered substitute implanted to his/her body, and later completely recover healthy functioning. Tissue engineering technologies utilize bio-fabricated scaffolds with cells and growth factors to provide high bio-functionality and biocompatibility for injury treatment (Chen, 2019), (Ikada, 2006). In 1993, the term tissue engineering was first documented (Vacanti, 2006) and after that, TE products, such as Organogenesis’ Apligraf that was used as a treatment for chronic wounds, started to be launched on the market (Jaklenec et al., 2012). Notably, in the decade from 1990 to 2000, over 70 TE companies worldwide spent more than $3.5 billion and grew at a compound annual growth rate of 16% (Lysaght and Reyes, 2001), indicating that commercialization was a critical success factor of the tissue engineering industry.

The tissue engineering market has grown rapidly in the past few years. In 2019, the global TE market was calculated to be around 9.9 billion US dollars, with an estimated compounded annual growth rate of 14.2% from 2020 to 2027 (Schachter, 2014; Yahya et al., 2021). At its early stage, TE was mainly featured by the development and application of biocompatible materials. Nowadays, it has been developed and evolved to be able to harvest cells from patients, multiply them in cell culture and then incorporate them into the biomaterials or scaffolds for treating orthopedic and musculoskeletal problems, and/or repairing damaged skin, cartilage, bone, spinal cords and other organs (Griffith and Naughton, 2002), (Yahya et al., 2021). Among these cells, stem cells have the capacity to both renew and differentiate to one or more types of specialized cells. Recently, bioprinting, a subcategory of additive manufacturing (AM), has been emerged to fabricate three-dimensional (3D) constructs such as cell-incorporated scaffolds, compartmentalized tablets, nano/micro carries for drugs and vaccines (Rajaram et al., 2012; Murphy and Atala, 2014; Moroni et al., 2018; Naghieh et al., 2018; Sadeghianmaryan et al., 2020; Tatara, 2020; Delkash et al., 2021; Ning et al., 2021; Malekpour and Chen, 2022; Mohabatpour et al., 2022; Sadeghianmaryan et al., 2022; Wang et al., 2022; Yazdanpanah et al., 2022). More recently, there are growing interests to apply bioprinting to combat infectious diseases (CID) including coronavirus disease 2019 (COVID-19) (Zimmerling and Chen, 2021), (Zimmerling and Chen, 2020), severe acute respiratory syndrome coronavirus (SARS), Ebola, middle eastern respiratory syndrome coronavirus (MERS) and Zika (Chen, 2019), (Tatara, 2020), (Bell, 2003; Gomes et al., 2014; Chang et al., 2016; Zimmerling et al., 2021). The increasing frequency of these infectious diseases means that new, rapid-responding, mechanisms/strategies for CID have high priority.

In light of the significant number of patients waiting for treatment of tissue/organ injuries every year and the increasing frequency of infectious diseases, there is a dire need for innovations to treat tissue/organ injuries and combat/control infectious diseases. For this, it is desired for researchers, innovators and policy makers to understand the current trends in the TE field so as to identify the needs and opportunities for supporting academic research activities and the pathway to commercialize new discoveries and technologies. In this work, we examined the current states and trends in this emerging field in Canada, and further identify the leading research on tissue engineering as well as its impactful commercialization ventures and economic growth. As per the previous market analysis conducted in the United States (Kim et al., 2019), we categorized the TE industry in Canada into four segments of *Bioprinting*, *Biomaterials*, *Cells and Biomaterials*, and *Stem Cells* for our analysis.

In addition to TE industry in Canada, we also examined to clinical trials since it was the critical step for new clinical technologies to develop medical treatments for now incurable diseases possible. The earliest clinical application of human cells in TE started in the 1980s for skin tissue (Ikada, 2006). Clinical tissue engineering products generally take a long time to develop but can be impactful and financially rewarding. It was an excellent chance to explore current states of tissue engineering research and clinical trials in Canada to understand upcoming entrepreneurial opportunities. In our work, we analyzed the funding resources and current phases of clinical trials available via public information. Taken together, our work provided an overall picture of current TE industries in Canada and potentially an insight into the global market.

## 2 Methods

### 2.1 Criteria for compiling the company list

In summer 2021, we searched and analyzed both public and private tissue engineering companies that were active in Canada. For each company, we collected online information, including company websites and Canada’s business registries website, and then analyzed and determined if they were active within 2011–2020 to the company list for examination. The international companies that had an executive office in Canada were also included in the list though their headquarters are not located in Canada.

Given the promise of bioprinting nowadays, we centered our efforts on commercialization activities of bioprinting in tissue engineering and regenerative medicine. We included stem cells banking institutes, which provided the key complementary technology enabler components such as stem cells. Companies focused on biomaterials were also included for a similar reason. Since our focus was the commercialization of innovative tissue engineering technologies, we included only companies that had commercialization of innovative technologies; while companies that were secondary dealerships and retailers were excluded from the list. Indeed, regenerative and reconstructive functions to damaged tissues were our key criteria to compile the company list, while the service and contract research organizations were excluded. Immunotherapies, genetic modeling, and cancer therapies were excluded as well because they were out of the scope of our definition. We excluded *in vitro* fertilization centers, the cosmetic industry, education, media, and financial service companies to keep the list focused on innovation-based TE companies.

We classified companies into four categories, i.e., Bioprinting, Biomaterials, Cells and Biomaterials, and Stem Cells. Companies involved in the bioprinting of products for healthcare and tissue engineering applications were classified in the “*Bioprinting*” category. Companies that predominantly produced materials for tissue engineering applications were classified in the “*Biomaterials”* category. Companies utilizing cells incorporated with materials for tissue engineering applications were classified under the “*Cells and Biomaterials*” category. Finally, companies that focused on altered or unaltered stem cells come under the “*Stem Cells*” category.

### 2.2 Gathering company data

Based on the criteria and keywords established above, we searched for company information and data from open internet resources. The public company details and status were tabulated using the official “*Search for a Federal Corporation*” tool via Corporations Canada website, from where the information including the registered address, directors’ name, and annual filings were collected. Details for private companies were gathered from their official websites and LinkedIn profiles. We gathered financial details and modeled values such as funding and sales of public companies from their annual reports and/or articles for private companies since they were not publicly available. However, exact details about the number of employees and specific funding were not collected. Products launched by the complied companies were searched and collected from their websites and classified into the four groups in terms of their functions for our analysis.

The data on clinical trials were publicly available in the National Library of Health and Health Canada’s Clinical Trials Database (H. C. Government of Canada, 2010). We examined the active clinical trials ongoing between October 2011 and July 2020 and excluded those under withdrawn and unknown status. The clinical trial data were also searched and obtained via the company’s websites and funding-related articles. Specifically, we searched the internet for companies and clinical trials between October 2011 and July 2020 using keywords such as “*tissue engineering*”, “*bioprinting*”, “*regenerative medicine*”, “*stem cells*”, “*biomaterials*”, “*scaffold*” and “*hydrogel*”. Additionally, we enabled google alerts for the keywords including “*bioprinting*”, “*tissue engineering*” and “*stem cells*” between April and June 2021 to update the new startups and details related to funding. The data collection was completed by July 2021, and the information available after July 2021 was not added. As such, the data presented in this paper may not be completed and are subject to further change with new trending innovations and inventions. Our results and findings represent the trends based on our data collected the companies within Canada.

## 3 Results

We identified in total 30 public and private companies and among them, 24 companies had their full details available. In general, there were approximately 3,000 employees in these companies involved, either directly or indirectly, in the tissue engineering field or market. These companies generated an estimated revenue of USD $67 million in the year 2020. The compiled list of companies can be found in Supplementary Appendix S1.

### 3.1 Locations of the tissue engineering companies

**FIGURE 1:**
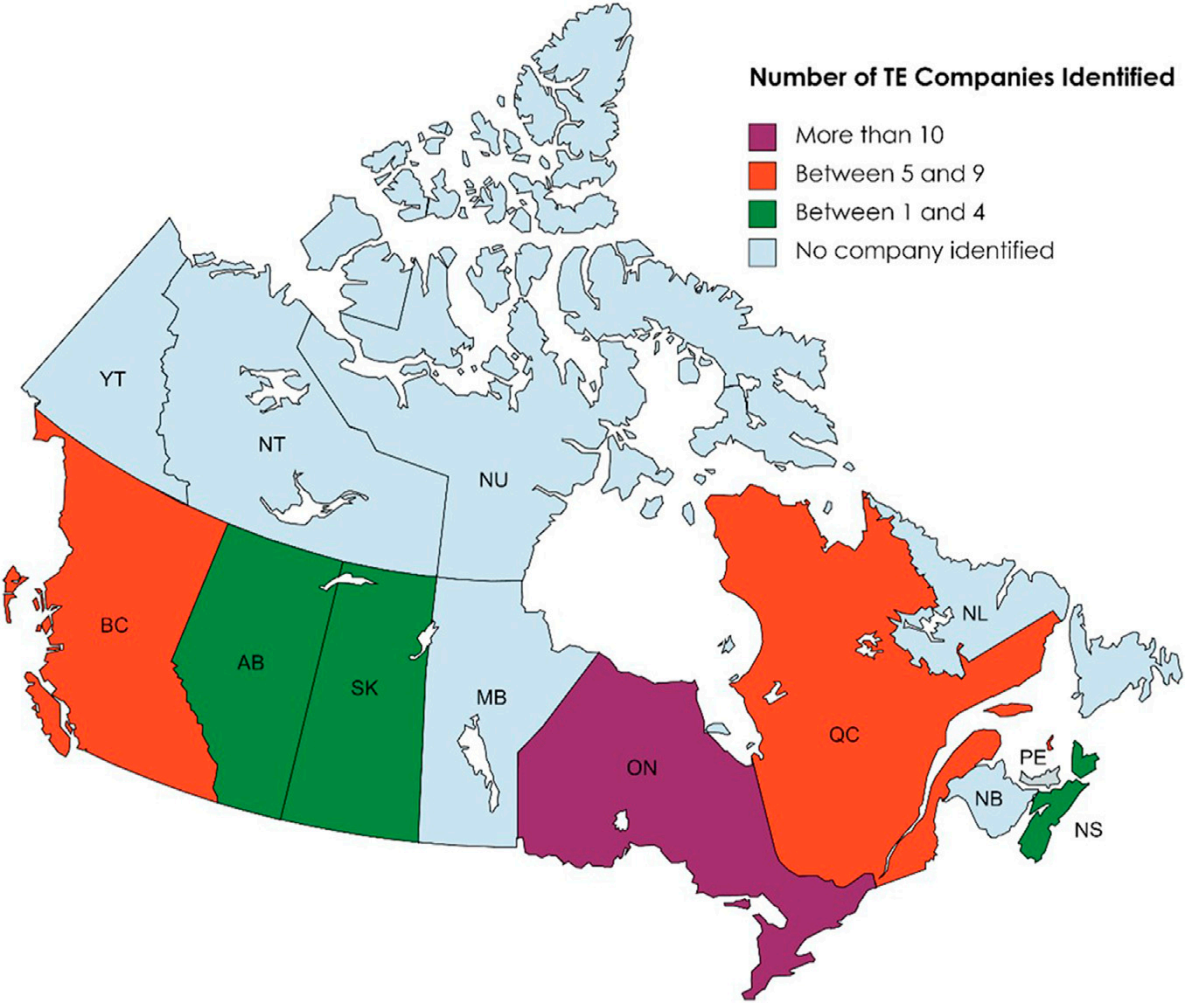
Number of TE companies in each province of Canada. Created with mapchart.net.

Figure 1 shows the geographical locations of TE company headquarters across Canada. Companies with headquarters oversea but executive offices in Canada were also included into the provinces where the executive offices are located. In this classification, we created four groups of provinces based on the number of TE companies in each province in an increment of five companies. Ontario had the highest number of headquarters and is the only province to have over ten companies examined in our study. Followed are British Columbia and Quebec, each had a number of companies between 5–10. The third group are the provinces with 1–5 companies, including Alberta, Saskatchewan, and Nova Scotia. No tissue engineering companies were identified in the rest of the provinces and territories based on our criteria and time frame.

### 3.2 Company size and revenues

**FIGURE 2:**
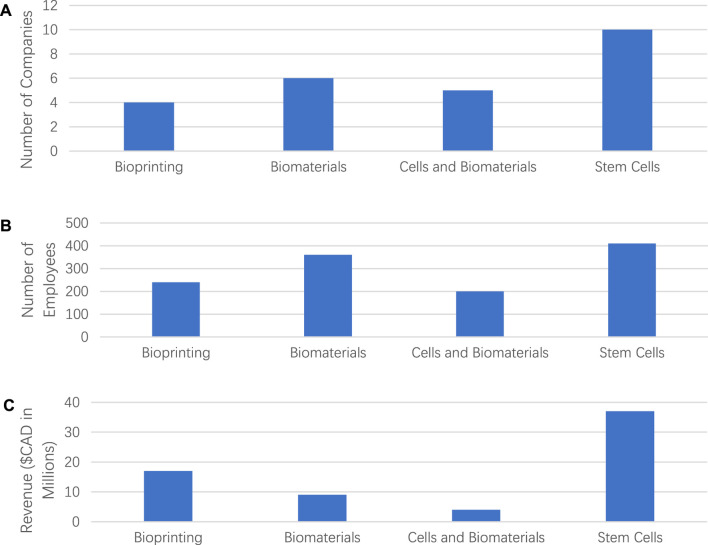
**(A)** Number of companies in each of analyzed categories, **(B)** number of employees employed by all the companies in each of the four categories, and **(C)** revenue generated by all the companies of four categories.

We examined and analyzed the size of companies and revenues for the four classified categories. As shown in Figures 2A, B, the category of “*Stem Cells*” had the most companies out of all four categories with more than 400 employees, followed by the Biomaterials category with more than 300 employees. On the other hand, the categories of “*Bioprinting”* and “*Cells and Biomaterials*” were relatively small scale with around 200 employees. On average, the category of “*Biomaterials*” had the most significant number of employees per company over 50. The number of employees was counted by only those directly involved in the companies, and third-party employees were not counted.

Figure 2C shows the revenues of tissue engineering products in each category. We counted the products only of commercial companies. The category “S*tem Cell*” generated the highest revenue compared to other categories. All the companies in “S*tem Cell*” category generated a total of revenue around USD$36 million, which was 56% of overall revenue in TE-related industries. One possible reason for the result was that most companies in other categories were under the clinical trial phase, with less revenue than the Stem Cell category. The category of “*Bioprinting*” generated up to USD$15.8 million, about 24% of total revenue. In addition, the categories of “*Biomaterials*” and “*Cells and Biomaterials*” generated revenue of USD$9 million and USD$4.5 million, respectively. However, the spending details were not recorded as they were unavailable for all categories.

### 3.3 Current clinical trials

**FIGURE 3:**
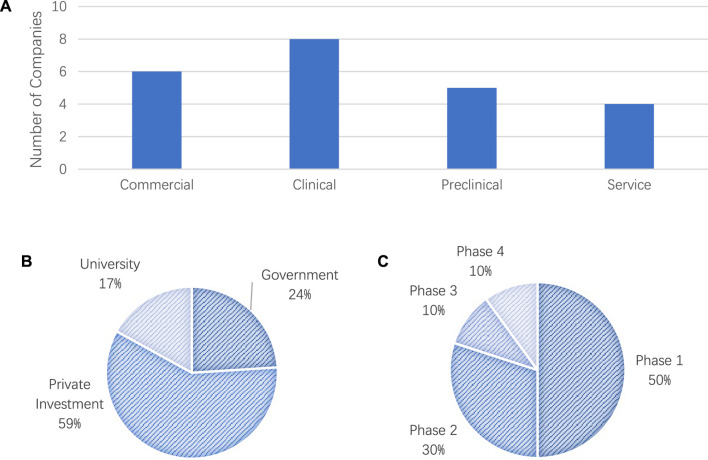
**(A)** Number of Companies in the four stages of commercialization: 1) commercia, 2) clinical, 3) preclinical, and 4) service, **(B)** Source of funding for clinical trials and **(C)** phases of clinical trials.

We also analyzed the current trends base do the collected data on clinical trials in the TE field. We collected and categorized the funding details into four stages, i.e., *Commercial, Clinical, Preclinical*, and *Service*. The total pre-clinical trials funding for the all the companies listed was around USD$283 million, where the category of “*Stem Cells*” received the highest funding. As shown in Figure 3A, most of the companies were currently in the stage of clinical trials.

Our analysis results on the funding resources demonstrated that more than 59% of the total TE-related clinical trials were being sponsored by industry. Our results also showed that there was continuous interest from industry in promoting TE products on the markets. In addition to the industry funding, 24% of the funding was provided by the government and 17% by universities as shown in Figure 3B. We also examined and investigated the status of the current phase of clinical trials for tissue engineering products with the results shown in Figure 3C. Around 50% of the clinical trials were in Phase 1 of the clinical trials, which suggests the potential increase in the number of new products undergoing clinical trials.

## 4 Discussion

While the reports have been published on tissue engineering industry with primary focus on the United States (Lysaght and Reyes, 2001), (Kim et al., 2019), (Hellman, 2008), (Parenteau, 2011), the work presented in this paper is the first attempt to examine the current development of tissue engineering industry in Canada, and to further establish methods that can be applied in analysis with more information becoming available in the future. With our collected information and/or data, we found similar trends in Canada as those in the United States, as reported in the previous studies (Lysaght and Reyes, 2001), (Kim et al., 2019), (Hellman, 2008), (Parenteau, 2011). Indeed, our results demonstrated that companies focused on stem cells generated the highest revenues and created the highest employment opportunities. Our results also shown that industry provided the majority of clinical trial funding similar to the United States. However, it is worthwhile to note that the government and universities provided a larger percentage of the funding compared to that of the United States. This may be due to the different investment structure in Canada, as well as the current stage of the commercialization of tissue engineering in Canada. Regardless, it might be worthy for innovators and entrepreneurs to investigate opportunities in the universities and public funded research institutes.

**FIGURE 4:**
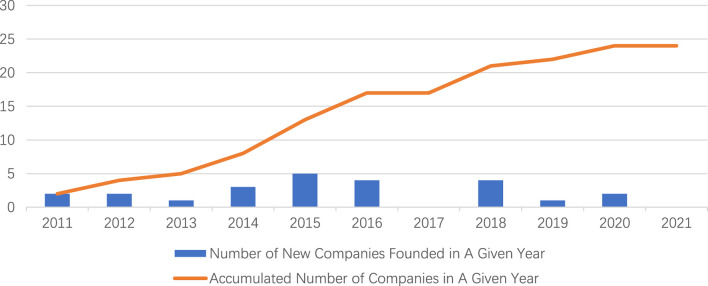
New TE companies founded Between 2011 and 2020.

In our study, we also mapped the company’s founding year and the origin of the companies. Out of the 24 companies identified, nine (9) of them were university spin-offs. Among these university-based companies, five (5) have obtained at least one patent for their technology, which is higher as compared to 4 out of 16 non-university-based companies. The number of new companies founded in each year between 2011 and 2020 were mapped in Figure 4, with more companies founded between 2011 and 2016. The trend for new companies remains steady since 2016, and potentially slowing down. It is worth for the innovators and policy makers to investigate and understand the support needed for this growing industry.

Inspiring findings in the category of Bioprinting are that the market of this category was emerging and that despite being a new area of the tissue engineering industry (as demonstrated by a small number of companies), Bioprinting companies employed the second highest number of employees and generated the second highest revenues. We believe this demonstrated the potential of commercialization and entrepreneurial opportunities of bioprinting related technologies in Canada. In our project, we identified four companies in Canada working on bioprinting based technology solution. These companies used dry-spinning and ink injection technologies; and two of the companies also provide solutions for biomaterials.

In the category for Biomaterials, a total of six companies were identified. Majority of the companies are still in the stage of clinical trials. These materials include chitosan, hyaluronan and other forms of hydrogels. Due to the nature of these early-stage companies, we were unable to collect further information regarding the detailed technology at this moment. However, several patents were identified with more information available.

Additionally, unlike that of the previous reports on the United States, our results illustrated that most of the companies in Canada were still in the stage of clinical trials rather than the commercialization stage. These results suggest that the tissue engineering industry is an emerging and upcoming opportunity in Canada. Meanwhile, it should be noted that the number of clinical trials presented in this paper, especially that of early stages, may not be able to accurately represent the future commercialization opportunities. As such, it is urged that following-up studies be needed to better understand the development of Canadian tissue engineering industry.

## 5 Conclusion

Tissue engineering aims to produce tissue/organ substitutes to treat damaged tissues/organs and tissue engineering industry has been emerging over the past decade in Canada. Our results shown there were a total of 25 companies established in Canada, which would continue to impact on the healthcare sector in combating infectious diseases and life-threatening disorders in the years to come. The growth of this field in future will not only focus on the innovations but also on the regulations of Health Canada, Food and Drug Administration, and other guidelines.

We identified two opportunities for innovators in relation to the tissue engineering industry. First, bioprinting is a promising direction that is worth innovators and entrepreneurs exploring. Second, universities and publicly funded research institutes may provide new technologies and products that are worth exploring.

In conclusion, this work provided an overview of tissue engineering industry in Canada up to June 2021. The established methods also can be applied and adopted in similar studies or analysis as new information becomes available in the future.

## Data Availability

The original contributions presented in the study are included in the article/Supplementary Material, further inquiries can be directed to the corresponding author.

## References

[B1] Bell D. (2003). Public health interventions and SARS spread, 2003. Emerg. Infect. Dis. 10 (11), 1900–1906. 10.3201/eid1011.040729 PMC3329045 15550198

[B2] Chang C. Ortiz K. Ansari A. Gershwin M. E. (2016). The Zika outbreak of the 21st century. J. Autoimmun. 68, 1–13. 10.1016/j.jaut.2016.02.006 26925496 PMC7127657

[B3] Chen X. B. (2019). Extrusion bioprinting of scaffolds for tissue engineering. Springer.

[B4] Cihi (2023). Organ replacement in Canada: CORR annual statistics | CIHI. Available at: https://www.cihi.ca/en/organ-replacement-in-canada-corr-annual-statistics (accessed May 07, 2023).

[B5] Delkash Y. Gouin M. Rimbeault T. Mohabatpour F. Papagerakis P. Maw S. (2021). Bioprinting and *in vitro* characterization of an eggwhite-based cell-laden patch for endothelialized tissue engineering applications. J. Funct. Biomater. 12 (3), 45. 10.3390/jfb12030045 34449625 PMC8395907

[B6] Gomes M. F. Pastore y Piontti A. Rossi L. Chao D. Longini I. Halloran M. E. (2014). Assessing the international spreading risk associated with the 2014 West African Ebola outbreak. PLoS Curr. 6. 10.1371/currents.outbreaks.cd818f63d40e24aef769dda7df9e0da5 PMC4169359 25642360

[B7] Griffith L. G. Naughton G. (2002). Tissue engineering--current challenges and expanding opportunities. science 295 (5557), 1009–1014. 10.1126/science.1069210 11834815

[B8] H. C. Government of Canada (2010), “Clinical trials Database (CTDB),”, . Aug. 25 Available at: https://health-products.canada.ca/ctdb-bdec/index-eng.jsp (accessed May 01, 2023).

[B9] Hellman K. B. (2008). Tissue engineering: Translating science to product. Top. Tissue Eng. 4, 1–28.

[B10] Ikada Y. (2006). Challenges in tissue engineering. J. R. Soc. Interface 3 (10), 589–601. 10.1098/rsif.2006.0124 16971328 PMC1664655

[B11] Jaklenec A. Stamp A. Deweerd E. Sherwin A. Langer R. (2012). Progress in the tissue engineering and stem cell industry ‘are we there yet? Tissue Eng. Part B Rev. 18 (3), 155–166. 10.1089/ten.teb.2011.0553 22220809

[B12] Kim Y. S. Smoak M. M. Melchiorri A. J. Mikos A. G. (2019). An overview of the tissue engineering market in the United States from 2011 to 2018. Tissue Eng. Part A 25, 1–8. 10.1089/ten.tea.2018.0138 30027831 PMC6352506

[B13] Lysaght M. J. Reyes J. (2001). The growth of tissue engineering. Tissue Eng. 7 (5), 485–493. 10.1089/107632701753213110 11694183

[B14] Malekpour A. Chen X. (2022). Printability and cell viability in extrusion-based bioprinting from experimental, computational, and machine learning views. J. Funct. Biomater. 13 (2), 40. 10.3390/jfb13020040 35466222 PMC9036289

[B15] Mohabatpour F. Duan X. Yazdanpanah Z. Tabil X. L. Lobanova L. Zhu N. (2022). Bioprinting of alginate-carboxymethyl chitosan scaffolds for enamel tissue engineering *in vitro* . Biofabrication 15 (1), 015022. 10.1088/1758-5090/acab35 36583240

[B16] Moroni L. Boland T. Burdick J. A. De Maria C. Derby B. Forgacs G. (2018). Biofabrication: A guide to technology and terminology. Trends Biotechnol. 36 (4), 384–402. 10.1016/j.tibtech.2017.10.015 29137814

[B17] Murphy S. V. Atala A. (2014). 3D bioprinting of tissues and organs. Nat. Biotechnol. 32 (8), 773–785. 10.1038/nbt.2958 25093879

[B18] Naghieh S. Sarker M. D. Izadifar M. Chen X. (2018). Dispensing-based bioprinting of mechanically-functional hybrid scaffolds with vessel-like channels for tissue engineering applications–a brief review. J. Mech. Behav. Biomed. Mat. 78, 298–314. 10.1016/j.jmbbm.2017.11.037 29197301

[B19] Ning L. Zhu N. Smith A. Rajaram A. Hou H. Srinivasan S. (2021). Noninvasive three-dimensional *in situ* and *in vivo* characterization of bioprinted hydrogel scaffolds using the X-ray propagation-based imaging technique. ACS Appl. Mat. Interfaces 13 (22), 25611–25623. 10.1021/acsami.1c02297 34038086

[B20] Parenteau N. L. (2011). “Commercialization of engineered tissue products,” in Advanced wound repair therapies (Amsterdam, Netherlands: Elsevier), 495–523.

[B22] Rajaram A. Chen X.-B. Schreyer D. J. (2012). Strategic design and recent fabrication techniques for bioengineered tissue scaffolds to improve peripheral nerve regeneration. Tissue Eng. Part B Rev. 18 (6), 454–467. 10.1089/ten.teb.2012.0006 22646535

[B23] Sadeghianmaryan A. Naghieh S. Alizadeh Sardroud H. Yazdanpanah Z. Afzal Soltani Y. Sernaglia J. (2020). Extrusion-based printing of chitosan scaffolds and their *in vitro* characterization for cartilage tissue engineering. Int. J. Biol. Macromol. 164, 3179–3192. 10.1016/j.ijbiomac.2020.08.180 32853616

[B24] Sadeghianmaryan A. Naghieh S. Yazdanpanah Z. Alizadeh Sardroud H. Sharma N. Wilson L. D. (2022). Fabrication of chitosan/alginate/hydroxyapatite hybrid scaffolds using 3D printing and impregnating techniques for potential cartilage regeneration. Int. J. Biol. Macromol. 204, 62–75. 10.1016/j.ijbiomac.2022.01.201 35124017

[B25] Schachter B. (2014). Therapies of the state. Nat. Biotechnol. 32 (8), 736–741. 10.1038/nbt.2984 25093881

[B26] Tatara A. M. (2020). Role of tissue engineering in COVID-19 and future viral outbreaks. Tissue Eng. Part A 26 (9–10), 468–474. 10.1089/ten.tea.2020.0094 32272857 PMC7249458

[B27] Vacanti C. A. (2006). The history of tissue engineering. J. Cell. Mol. Med. 10, 569–576. 10.1111/j.1582-4934.2006.tb00421.x 16989721 PMC3933143

[B28] Wang R. Deng S. Wu Y. Wei H. Jing G. Zhang B. (2022). Remodelling 3D printed GelMA-HA corneal scaffolds by cornea stromal cells. Colloid Interface Sci. Commun. 49, 100632. 10.1016/j.colcom.2022.100632

[B29] Yahya E. B. Amirul A. A. H.p.s. A. K. Olaiya N. G. Iqbal M. O. Jummaat F. (2021). Insights into the role of biopolymer aerogel scaffolds in tissue engineering and regenerative medicine. Polymers 13 (10), 1612. 10.3390/polym13101612 34067569 PMC8156123

[B30] Yazdanpanah Z. Johnston J. D. Cooper D. M. Chen X. (2022). 3D bioprinted scaffolds for bone tissue engineering: State-of-the-art and emerging technologies. Front. Bioeng. Biotechnol. 10, 824156. 10.3389/fbioe.2022.824156 35480972 PMC9035802

[B31] Zimmerling A. Chen X. (2020). Bioprinting for combating infectious diseases. Bioprinting 20, e00104. 10.1016/j.bprint.2020.e00104 33015403 PMC7521216

[B32] Zimmerling A. Chen X. (2021). Innovation and possible long-term impact driven by COVID-19: Manufacturing, personal protective equipment and digital technologies. Technol. Soc. 65, 101541. 10.1016/j.techsoc.2021.101541 36540655 PMC9754673

[B33] Zimmerling A. Zhou Y. Chen X. (2021). Bioprinted constructs for respiratory tissue engineering. Bioprinting 24, e00177. 10.1016/j.bprint.2021.e00177

